# Change in Quality of Life After the Relocation of a National Forensic Hospital: A Dundrum Forensic Redevelopment Evaluation Study (D-FOREST)

**DOI:** 10.1192/j.eurpsy.2024.1202

**Published:** 2024-08-27

**Authors:** A. O’Reilly, M. U. Waqar, M. U. Iqbal, H. Amin, H. G. Kennedy, M. Davoren

**Affiliations:** ^1^Central Mental Hospital, National Forensic Mental Health Service, Portrane; ^2^Dundrum Centre for Forensic Excellence, Trinity College Dublin, Dublin, Ireland

## Abstract

**Introduction:**

Forensic psychiatric services address the therapeutic needs of mentally disordered offenders in a secure setting. Clinical, ethical, and legal considerations underpinning treatment emphasize that the Quality of Life (QOL) of patients admitted to forensic hospitals should be optimised.

**Objectives:**

This study aims to examine changes in the QOL in Ireland’s National Forensic Mental Health Service following its relocation from the historic 1850 site in Dundrum to a new campus in Portrane, Dublin.

**Methods:**

This multisite prospective longitudinal study is part of the Dundrum Forensic Redevelopment Evaluation Study (D-FOREST). Repeated measures were taken for all inpatients in the service at regular six-monthly intervals. The WHOQOL-BREF questionnaire was offered to all inpatients and an anonymised EssenCES questionnaire was simultaneously used to measure atmosphere in the wards. Data were obtained at five time points for each individual patient and ward. WHOQOL-BREF ratings were obtained across five time points with comparisons for four time intervals, including immediately before and after relocation. For 101 subjects across the four time intervals, 215 sets of data were obtained; 140 before and 65 after relocation with 10 community patients who did not move. Using Generalised Estimating Equations (GEE) to correct for multiple comparisons over time, the effect of relocation, with community patients as a control, was analysed by ward cluster and whether patients moved between wards. Observations were categorised according to security level — high dependency, medium secure, rehabilitation, or community — and trichotomised based on positive moves to less secure wards, more secure wards (negative moves), or no moves.

**Results:**

The hospital’s relocation was associated with a significant increase in environmental QOL (Wald X^2^=15.9, *df*=1, *p*<0.001), even when controlling for cluster location, positive and negative moves. When controlling for ward atmosphere, environmental QOL remained significantly increased after relocation (Wald X^2^=10.0, *df*=1, *p*=0.002). EssenCES scores were obtained within the hospital for three time points before relocation and two time points afterward. No significant differences were found in the three subscales before and after the relocation. All three EssenCES subscales progressively improved with decreasing security level (Patient’s Cohesion: Wald X^2^=958.3, *df*=1, *p*<0.001; Experienced Safety: Wald X^2^=152.9, *df*=5, *p*<0.001; Therapeutic Hold: Wald X^2^=33.6, *df*=3, *p*<0.001).

**Conclusions:**

The GEE model showed that the hospital’s relocation improved self-reported environmental QOL. The cluster location made significant differences, as expected for a system of stratified therapeutic security, with a steady improvement in scores on all three atmosphere subscales.

**Disclosure of Interest:**

None Declared

